# Clinicians’ knowledge and attitudes towards patient reported outcomes in colorectal cancer care – insights from qualitative interviews

**DOI:** 10.1186/s12913-021-06361-z

**Published:** 2021-04-20

**Authors:** Nora Tabea Sibert, Christoph Kowalski, Holger Pfaff, Simone Wesselmann, Clara Breidenbach

**Affiliations:** 1 German Cancer Society, Kuno-Fischer-Straße 8, 14057 Berlin, Germany; 2 University of Cologne, Faculty of Human Sciences, Faculty of Medicine and University Hospital Cologne, Institute of Medical Sociology, Health Services Research and Rehabilitation Science, Eupener Str. 129, 50933 Köln, Germany

**Keywords:** Patient-reported outcomes, Patient-reported outcome measures, Implementation, Routine care, EORTC, Cancer care

## Abstract

**Introduction:**

Patient-reported outcomes (PROs) can be used in cancer care to monitor patients’ disease-related symptoms and functional status. However, successful implementation of such instruments is only possible if clinical staff are convinced of the clinical benefits. It is therefore crucial to investigate the attitudes of clinical staff to PROs in routine cancer care.

**Methods:**

Semi-structured, guideline-based interviews were held with 12 clinicians working in certified colorectal cancer centers in Germany who are taking part in an observational study on PROs (five surgeons, two oncologists, one psycho-oncologist, two oncological care nurses, one stoma therapist, and one physician assistant) in order to investigate firstly, how clinicians describe PRO instruments (“wording”); and secondly, the clinicians’ general attitude toward PROs. A qualitative content analysis according to Kuckartz was performed.

**Results:**

The wording used to describe PROs was not consistent. Statements on attitudes toward PROs were very heterogeneous and were therefore categorized into “(rather) positive” and “(rather) negative.” The principal advantages of PROs mentioned by participants included broader, structured knowledge about patients and treatment, as well as relevance for patients. Subcategories for (rather) negative attitudes included statements expressing doubts about the questionnaires and “no need for PROs.”

**Discussion:**

The clinicians participating mainly expressed fairly positive attitudes toward PROs. However, they had little knowledge about PROs in general and the interviews therefore mainly reflect their expectations and assumptions about them. These initial impressions may be regarded as providing a basis for future implementation strategies and for training of clinicians on how to use PROs in routine cancer care.

**Supplementary Information:**

The online version contains supplementary material available at 10.1186/s12913-021-06361-z.

## Introduction

Cancer care is a prolonged and multidisciplinary task. Cancer patients often need not only definitive treatment, such as surgery, but also a more complex treatment strategy involving surgery, chemotherapy, and radiotherapy, or a combination of these. The various treatment pathways have different, but often severe side effects. As a result, health-related quality of life and functional status are often severely affected by oncological disease and may vary substantially with the treatment regimen chosen, the treating clinicians, and over time. Monitoring health-related quality of life and functional status regularly is therefore an important part of cancer care [1].

Traditionally, this monitoring involves the clinical staff taking the patient’s history and carrying out medical assessments such as blood tests or medical imaging. Nevertheless, there is evidence that clinicians underestimate the symptom burden and adverse effects [2]. Standardized questionnaires filled out by the patients themselves are therefore instruments that can help clinical staff to identify disease and treatment-specific problems effectively. These instruments are called patient-reported outcomes (PROs), and many clinical trials have already shown that they have benefits for monitoring purposes [3]. To allow close monitoring of each case, the patients complete PRO questionnaires, and their answers and results are passed on to the clinicians treating them (and sometimes to the patients themselves). Using these results, clinicians can assess and reevaluate treatment choices where necessary and assess signs of disease progression. Several clinical trials that have focused on regular monitoring of patients’ functional status using PROs have reported improvements in overall survival [4] [5]. In Germany, for example, Klinkhammer-Schalke et al. could show in a randomized clinical trial a systematic monitoring of PROs and – based on a so called “quality of life (QoL) profile” – initiating personalized treatment options can enhance routine colorectal cancer care. In this setting, PROs were collected using the EORTC QLQ-C30 and -CR29 questionnaires and summarizing specific scores to a set of 13 QoL domains based on an expert consensus. If a patient scored lower than a pre-defined threshold, their physicians were advised to react therapeutically (e. g. by offering a specialized pain treatment or psycho- oncological assistance) [6]. Implementing PROs in routine cancer care can therefore help clinical staff identify disease-related problems in cancer patients, and the patients can consequently receive more personalized therapy.

However, PROs have not yet become fully established in routine cancer care, and most clinical staff do not yet have any initial experience with them. In order to successfully implement PROs in routine clinical work, clinicians — oncologists, surgeons, nurses, and psychotherapists — need to be involved as early as possible. The “knowledge-to-action” (KTA) framework described by Graham et al. [7] has previously been used for PRO implementation [8]. Key parts of the KTA framework are the knowledge creation – with its main aim to “tailor knowledge” - and the iterative action cycle. This action cycle reflects the processes needed to cause change in the behavior and attitudes of persons concerned. Key parts of the KTA action cycle are “identification of the problem and review and selection of knowledge”, “adaption of knowledge to local context”, “assessment of barriers to knowledge use”, “selection, tailoring and implementation of interventions”, “monitoring knowledge use”, “evaluation of outcomes” and “maintenance of knowledge use” [7]. Thus, the KTA framework can be used to identify crucial steps during the implementation process and has already been used in more than 140 implementation studies [9]. To identify appropriate implementation strategies, it is crucial to know whether and in what ways clinicians regard PROs as a useful tool for routine cancer care [10]. Eccles et al. have reported that Ajzen’s theory of planned behavior [11] can be successfully used to analyze changes in clinicians’ attitudes [12]. In addition to subjective standards and perceived behavioral control, an individual’s attitude toward a specific behavior is a major predictor for changes in behavior, according to Ajzen. The theory can also be adapted for research questions regarding implementation strategies, and information about the attitudes of the individuals affected by a specific behavior should be collected. In this context, “attitude toward a behavior” is taken to mean “the degree to which a person has a favorable or unfavorable evaluation or appraisal of the behavior in question” [13]. Ibidem, “perceived behavioral control” is explained as “people’s perception of the ease or difficulty of performing the behavior of interest” and thus can be analysed by exploring perceived facilitators and barriers for the use of PROs. The aims of the study were to 1) collect information about clinicians’ general attitudes toward and knowledge about PROs in routine cancer care, and 2) identify potential factors facilitating or inhibiting the use of PROs in practice. This analysis addresses the first aim. Results from the second aim will be published in a subsequent paper.

## Material and methods

### Study design

For the “Ergebnisqualität bei Darmkrebs: Identifikation von Unterschieden und Maßnahmen zur flächendeckenden Qualitätsentwicklung”, (EDIUM) study, clinicians’ from colorectal cancer centers (CCCs) certified by the German Cancer Society used the PRO questionnaires “European Organization for Research and Treatment of Cancer” (EORTC) QLQ-C30 [14] and EORTC QLQ-CR29 [15] to inquire about the functional status of colorectal cancer patients before the start of treatment and 1 year afterward. In Germany, approximately half of all newly diagnosed colorectal cancer patients are treated in a CCC certified by the German Cancer Society [16]. Thus, cancer care in CCCs is an important part of routine cancer care in Germany. The aim of the EDIUM study was to assess and compare the quality of care between certified CCCs in Germany using PROs. It is possible, but not compulsory, for the participating CCCs to use their patients’ baseline PROs for clinical decision-making. The study coordinators did not mandate which EORTC domains should be used nor thresholds for PROs if they were embedded in clinical decision-making because there is a lack of evidence regarding which EORTC domains are superior to others in colorectal cancer care [17]. However, the study coordinators provided a clinical guide on how to interpret the PRO results using the EORTC QLQ-C30 reference values manual [18]. Some CCCs therefore include PROs in decision-making processes, whereas others only use the PROs for comparison/benchmarking purposes. Nine months after the start of the study, clinicians from the participating CCCs were asked to take part in qualitative semi-structured interviews regarding their general attitudes to and initial experience with the PROs used in the study. The study was approved by the ethics committee of the Medical Association of Berlin (*Ärztekammer Berlin*) as part of the EDIUM study (eth-19/18) and was funded by the Innovation Fund of the Federal Joint Committee (G-BA).^1^

### Recruitment of participants

Interview participants were recruited from the CCCs participating in the EDIUM study using a mix of convenience and snowball sampling approaches: The study administrators in the participating CCCs were informed about the interviews and could nominate clinicians who were willing to answer questions about their use of PROs. Eligible interview participants had to be involved in in-patient colorectal cancer care in one of the centers — such as oncologists, surgeons, nurses, or psychotherapists. However, they did not have to be actively involved in the EDIUM study; only the center they were working for needed to be enrolled in the study. Nevertheless, study centres were asked during the sample recruitment process if they used PROs collected for EDIUM for clinical decision making. Besides experience with PROs, sample saturation criteria also included profession and professional experience, participants’ gender and age, as well as the academic status of the CCCs they were working for. All of the participants provided written informed consent.

### Data collection

The interviews were conducted by C.B. or N.T.S., both female and aged between 25 and 27, and took place in the CCCs the clinicians were working for in November and December 2019. All of the interviews followed the same guideline (see additional file 1) and consisted of two parts. In each interview, the participants were provided with copies of the PRO questionnaire used for the EDIUM study. To begin with, the participants were asked about their profession and position in cancer care in their center, as well as their involvement in EDIUM. Questions about their general knowledge about PROs (including clarification of wording) followed. If the participants had not previously heard the term “PRO,” the basic concept was briefly described to them and examples were given. The participants were then asked about their attitude toward PROs in general and specifically those used for the EDIUM study. Factors facilitating or inhibiting the use of PROs in cancer care were inquired about as well, but these are reported elsewhere. In addition, if participants were in favor of the clinical use of PROs, they were asked about which part of cancer care and at which time point they would include PROs in routine cancer care.

In the second part of the interview, different options for presenting PRO scores were discussed. For this purpose, five different approaches were presented to each participant. The results of this second part are reported elsewhere. The interviews took place in the centers where the participants were working and were conducted in German. All of the interviews were audio-recorded and transcribed in an anonymized format. The guideline and the quotations from the participants’ responses used in this article were translated from German by a professional translation office.

### Data analysis

The transcribed interviews were analyzed using Kuckartz content analysis [19]. Both interviewers (C.B., N.T.S.) started by coding the first three interviews deductively, using the main categories “wording,” “general attitude,” “data collection,” “clinical use,” and “result presentation,” and developed additional categories and subcategories inductively. For the research question, N.T.S. then developed a code book using the main categories “wording” and “general attitude,” in collaboration with C.B., and a qualitative content analysis of the resulting categories was performed. In a second step, differences in the perception of PROs among the various specialties and professions represented were investigated (referred to as group comparison) in order to explore to what extent attitudes towards PROs varied across professional affiliation. Transcription and data analysis were carried out with the help of the software programs f4transcript and f4analysis, version 2.5.4.

## Results

### Sample

Twelve clinicians from eight CCCs were interviewed: five surgeons, two oncologists, one psycho-oncologist, two oncological care nurses, one stoma therapist, and one physician assistant. Their ages ranged from 31 to 58 years. Participants were eligible to participate regardless of the duration of their professional experience. For details, see Table 1.

**Table 1:** Interview sample: 12 participants from eight colorectal cancer centers

Profession		
Physicians		7
Surgery	5	
Internal medicine	2	
Nurses		4
With oncological specialization	2	
Physician assistant	1	
Stoma therapist	1	
Psychotherapist		1
Gender		
Female		9
Male		3
Age: mean (SD)		46 (9)
Colorectal cancer centers		
In a university hospital		2
Not in a university hospital		6

### Qualitative content analysis

The main categories (referred to as “themes”) are described below, along with results from the interviews. The code book, with category definitions and anchor examples, is presented in additional file 2.

#### Theme 1: wording

At the beginning of the interview, each participant was asked what they called the questionnaires used for the EDIUM study or similar instruments. The way in which participants referred to PROs during the course of the interview was also analyzed. The participants mostly did not distinguish between the actual instrument and the results when talking about PROs. PROs were consequently mainly referred to as “(result of the) questionnaire.” Some of the participants indicated their understanding of the results of PROs by referring to them as “(functional or symptom) scales” (as proposed by the EORTC) or simply “quality of life.” Three participants claimed not to have any specific term for PROs (“No, we don’t have a name for it. Maybe we should think about one,” CH03f), and only two participants actually used the term “patient-reported outcomes.”

#### Theme 2: general attitudes toward PROs

In this category, the participants’ attitudes toward PROs were analyzed, including their thoughts on general possible uses for PROs. Their statements were classified into “(rather) positive attitude” and “(rather) negative attitude,” following the bipolar conception of attitude of Ajzen’s theory of planned behavior [11], and further information explaining the attitude was specified using subcategories.

#### Theme 2a: positive attitudes toward PROs

Statements that mainly highlighted possibilities and advantages of (clinically use of) PROs were classified as “(rather) positive.”

The participants mentioned three main possible uses. Firstly, many statements suggested that PROs could be used in a clinical setting:

- “I can imagine using this [the PRO instruments], or even having it included in treatment planning.” (CH01m)

The participants described different purposes and parts of cancer care for which PROs could be used clinically. To begin with, using PROs as a screening tool was mentioned:

- “If we weren’t seeing all of the patients personally, then I could imagine the questionnaire could also be quite useful for filtering out where we should go.” (PO01w)

The participants were also able to imagine using PROs for “treatment planning” and for “treatment monitoring”:

- “But I’d mainly be interested first of all in the questionnaire from beforehand, because from that you can quickly find out a lot of things you need to pay attention to, for treatment planning as well.” (IM01m)
- “So of course you could pass this questionnaire back to the therapist, that would be an option.” (PA0102)

In addition to clinical uses, “scientific use” of PROs — reflecting their current use in the EDIUM study — was also mentioned:

- “That’s why I think the thing with the questionnaires is very, very good, and of course — and this is where the scientific approach comes in — you can also quantify it.” (CH02f)

Thirdly, some participants referred to the “quality assurance” aspects of PROs:

- “These are different quality data from the purely key figures provided during the certification procedures, so to speak, and together it also produces a much more specific picture of the center.” (CH01m)

Nevertheless, these different usage possibilities of PROs mentioned by the participants were not always clearly distinguishable, and some statements remained ambiguous, highlighting the multipurpose approach of PROs:

- “Because I think you’d want to pass it back. Whether to individuals or just to a larger group, you’d have to see.” (PA0102)

Participants mentioned several major advantages of using PROs. To begin with, many of the aspects mentioned can be summarized using the subcategory “additional information gained”:

- “Personally, I’ve actually already noticed that you do also find out a few things about the patients using questionnaires like this that you wouldn’t otherwise get out, even if you have a long discussion with them.” (IM01m)
- “A whole lot of things may possibly come out in the process that we might not grasp like that as therapists at all.” (PA0102)

The opportunity to “visualize disease progression” through routine and periodic use of PROs was also mentioned:

- “But I just think, if you just follow it up over the course, and supposing these questionnaires become established now, maybe at each follow-up appointment, then I’d have a course like that and I could see whether it’s getting better or worse, and then it would actually help, I think. Then for the next check-up, I’ll somehow know, ‘Ah, last time he was doing worse, now I’ll need to take a more careful look today.’” (CH02f)

The “relevance to patients” of PROs was also discussed by some of the participants:

- “I think it’s very positive, because it’s relevant for the patients. I mean, so if we say we’ve had a curative treatment approach, then the patients will have to or may be able to live with the situation for another 10, 20, 30 years. And that needs to go along with a reasonable quality of life — and particularly with rectal cancer patients, both of us know that isn’t always the case.” (CH02f)

For some of the participants, the opportunity to use PROs in face-to-face discussions with patients, or as a way of screening for communication needs, was important (“support for patient–clinician communications”):

- “And then you can also use it to offer a discussion or to arrange for someone who could then offer a discussion.” (IM01m)

Finally, “standardization/quantification” was also highlighted as an advantage with PROs:

- “Because in the end it’s a matter of standardization, so that things can be made comparable.” (CH01m)

#### Theme 2b: negative attitudes toward PROs

Statements that tended to express doubts about PROs were categorized as “(rather) negative.” Inductively, two subcategories of these statements were found: “doubts about questionnaire” and “doubts about the need for PROs.”

The reasons that participants gave for having concerns about the questionnaires were manifold and sometimes contradictory. On the one hand, some participants complained about “unspecific questions” in the EORTC QLQ-C30 and -CR29:

- “Otherwise I think some of the others are also very unspecific and may not give you any concrete help … These are pretty standardized questionnaires … so to that extent you need to see what the analysis actually shows, what’s useful and what isn’t.” (CH04f)

By contrast, other participants were concerned about there being too many questions (“questions too specific”):

- “So in that sense for colorectal patients, I think it would actually be quite good to focus a bit on the typical symptoms, such as dry mouth, hair loss is maybe more to do with chemotherapy and not so much before the operation, that might be something I would tend to leave out. And maybe a few things could be summed up a bit. Pain here, for example, there are several forms here like abdominal pain, anal, rectal, and the incontinence stories could maybe tend to be summed up a bit.” (CH02f)

In addition, there were participants who tended to the view that the “questions are not relevant for in-patient cancer care”:

- “Well, I think that’s a problem of course for an acute-care hospital like this one … When the patients are back home, of course, and everything has settled down. I think then the whole thing becomes even more important again … But here [i.e. treatment in the center] it’s still a ‘worst case’ situation for lots of patients. And then of course it’s difficult to discuss things like that with them.” (PF01f)

The algorithm for scoring the questionnaire was also questioned (“unspecific scaling”):

- “What bothers me a bit, I mean, the scales, they’re not very differentiated at all, are they? … And I think the scaling isn’t very detailed. I mean, it’s actually relatively rough in my view.” (PO01w)

The participants gave a few reasons for doubting the need for PROs in cancer care, some of them alongside the advantages mentioned above. The main reasons mentioned can be summed up as “no additional information” being provided when using PROs:

- “Lots of the things are the same as what you have in routine clinical work anyway. Especially if the patients are in surgery or gastroenterology, then it’s hair loss, abdominal pain, anorectal pain, lots of things that are part of normal data collection, if I can put it like that … So that’s why I think it’s not really that relevant at the moment, because it’s actually in parallel with it, or maybe there’s some overlapping. So of course you could pass this questionnaire back to the therapist, that would be an option. So it’s already a bit superfluous.” (PA0102)

Some of the participants also emphasized that PROs “cannot replace face-to-face conversation” between clinicians and patients:

- “Well, it’s always — when it’s about very specific, personal areas, I think, then it’s, for many patients it gets difficult, it’s the financial side on the one hand, where many people probably find it hard to make a statement, that’s probably better done in a personal conversation, I think.” (IM01m)

### Comparison of professional groups

Overall, the different professional groups (physicians, nurses, psychotherapist) did not express entirely contrasting views. The physicians who were interviewed tended to describe multiple possible uses of PROs more often: they not only mentioned their clinical use, but also scientific benefits and opportunities for quality assurance provided by PROs. By contrast, the nurses who were participating mainly mentioned aspects of practical usage of PROs in routine clinical work.

It is also interesting that some of the participants tended to envisage possible clinical uses of PROs in specialties other than their own clinical work. Several participants, who were all involved in in-patient cancer care in CCCs, suggested that PROs could be useful for outpatient cancer care:

- “Well in the end, of course, the follow-up physician … er … would certainly also be a target, who’s still looking after them, because you already get a functional picture, and you should really take a look at that, because patients don’t always tell you everything, I mean just during the course of the follow-up, I think it can already, it can ask about relevant points, and the patient really has to properly write it down.” (CH01m)

It also appeared that specific professions were mentioned as the main intended users of PROs — e.g., psychotherapists or stoma therapists:

- “I mean, stoma therapists can benefit from it. It would be helpful for stoma therapists. It would be helpful for the psycho-oncologists, and in the end it would certainly be helpful for the physician providing further treatment.” (CH04f)

Despite this, neither the psychotherapist nor the stoma therapist who were interviewed expressed any need for PROs for their routine cancer care:

- “And also, we visit each patient personally. In other words, I have a conversation with every patient. In more or less detail, depending on the situation. That’s the one side that I have, I mean the direct patient contact. And on the other, I then also have a look at the EDIUM questionnaire and notice I already know the answers given in the questionnaire, because I had the personal contact and talked to the patients about their condition and everything troubling them and so on. I do also try to go through it and see if there’s anything to do, but at the moment I don’t get very much new information from it.” (PO01w)

### Summary: overview of clinicians’ fears and hopes with PROs

**Fig. 1:**
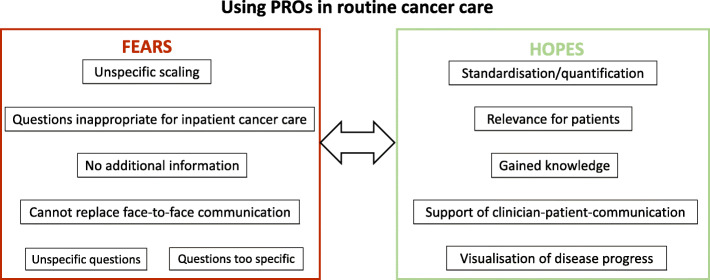
Fears and hopes expressed by participants regarding the use of patient-reported outcomes (PROs) in routine cancer care

At the time of the interviews, the participating clinicians showed little or no experience with PROs in routine cancer care. Their statements can therefore be regarded as reflecting their fears and hopes with PROs. Due to the limited experience the clinicians had with PROs, the statements they gave are partly inconsistent and ambiguous for some of the participants. Figure 1 encapsulates these fears and hopes as a synthesis of the reports.

## Discussion

Most of the participating clinicians were able to identify advantages provided by PROs in clinical use. Most of these advantages were seen in using PROs as a structured approach to quantifying cancer-related symptoms and functions, and hence to obtain more information about cancer patients and their treatment. The participants envisaged several use cases for PROs — not only as a clinical instrument, but also as an important scientific indicator of outcomes and for quality assurance. The multiple use cases may be very helpful in future efforts to successfully implement PROs in clinical contexts, as they highlight several strengths of PROs. If PROs are successfully implemented, they may make it possible to avoid multiple documentation of outcomes for different purposes in CCCs (e.g., certification, clinical documentation, observational or clinical studies). In combination with the quantifying aspects of PROs, this structuring and information bundling may help CCCs to work more effectively.

However, the clinicians participating in the present study mentioned reasonable doubts concerning PROs, including criticism of the questionnaire and scepticism regarding the need for PROs in clinical use. These doubts highlight the importance of involving clinicians as early as possible in processes of implementation when planning to use PROs in CCCs. To begin with, clinicians can contribute their specialist knowledge to the development of appropriate instruments. For the EORTC, for instance, this was an important part of the questionnaire development process from the beginning: for each questionnaire, different professional specialists are involved in developing relevant disease-related quality of life questionnaires [20]. However, clinicians’ involvement should not end with questionnaire development. If PRO scores tend to be too unspecific for some clinicians or departments, some authors have suggested using the item responses or including additional questionnaires for some indications [21, 22]. On the other hand, there are several implementation strategies that restrict the PRO scores to only the most relevant ones for a particular case [23].

Another important reason for the clinicians’ doubts might be their uncertainty of possible clinical consequences of PRO results - like therapy modification or even precise therapy options. Thus, clinicians remain uncertain about the practical usefulness of PROs – although there is already evidence indicating that PROs can serve as predictors of survival of especially colorectal cancer patients [24, 25]. Our results highlight that knowledge of how important PROs can be for colorectal cancer care has yet not reached clinicians’ treating colorectal cancer patients. In the late 1980s, Davis developed the “Technology Acceptance Model” (TAM) to describe how and if users accept and use technology [26]. Since then, TAM has widely been used in health care sciences, as well [27]. The “perceived usefulness” is an important factor for technology acceptance according to TAM and is defined as “perception that using system leads to enhanced personal performance” [27]. For implementing PROs, hence, concrete clinical implications or therapeutic options conditional upon particular PRO results could help to increase clinicians’ opinion about the usefulness of PROs. For example, Klinkhammer-Schalke et al. measured PROs in colorectal cancer patients and proposed tailored patient pathways, as described in the introduction. For patients with PRO results above or below preassigned thresholds, particular therapy options were proposed to clinicians [6]. However, it remains unclear how these thresholds were determined. Thus, thresholds and clinical implications should be developed together with clinicians as early as possible and thus could enhance clinicians’ acceptance of PROs.

In Germany, certified cancer centres are an important part in colorectal cancer care with approximately half of all colorectal cancer patients being treated in a CCC [16]. Regarding PROs, there is little to no experience in routine cancer care in Germany so far and PROs are only used in trials and outside routine care. For reaching as many colorectal cancer patients as possible in Germany, any attempts for implementing PROs in routine colorectal cancer care should involve cancer care in CCCs. From the findings of this analysis some practical implications might be derived: By showing that there is a lack of experience working with PROs in clinical-decision making which might trigger fears and hopes in clinicians, the present study supports the need to develop successful implementation strategies for PROs, taking clinicians’ concerns and ideas into account from the very start. For the contextualization of our findings, we used the KTA framework by Graham et al. [7]. It emphasizes the importance of active collaboration among all stakeholders. In a systematic review of factors facilitating and inhibiting the implementation of PROs, Foster et al. also highlight the importance of involving clinicians at an early stage [28]. Following the KTA framework’s action cycle as described above, our results on clinicians’ attitude towards PROs are an important prerequisite when closing the knowledge-practice gap and should be used when developing concrete PRO implementation strategies for the CCCs (referred to as “adapting the knowledge to the local context” within the KTA framework). For instance, it seems reasonable to organize training events (e.g., webinars, question-and-answer sessions) for professionals as early as possible in order to enhance clinicians’ knowledge about PROs [29]. The present findings may suggest possible aspects that should be discussed with clinicians in such training sessions. As Fig. 1 shows, the fears and hopes identified among clinicians are often parallel. Discussing these can thus enable clinicians to prioritize the purposes for which they want to use PROs. Moreover, results from the analysis of facilitators and barriers of PRO use in routine colorectal cancer care in our setting, as described in detail by Breidenbach et al., highlight the importance of a robust and easily accessible technical PRO infrastructure, as well as some organizational aspects (e. g. clear responsibility/coordination of PROs within the CCC or precise dissociation from other clinical monitoring tools). This “assessment of barriers to using knowledge” is another important phase of the KTA framework’s action cycle addressed in this study. With this knowledge about the prerequisites of PRO implementation in German CCCs, stakeholders like clinicians, as well as coordinators of CCCs or even medical societies as supporters, can start to tackle the next action cycle’s phase (“selection, tailoring and implementation of interventions”) with e. g. educational training as proposed above.

Our findings, however, are not only crucial when applying the KTA framework’s action cycle, but also highlight knowledge gaps in PROs research which should be approached with more emphasis (“knowledge creation” within the KTA framework): The clinicians’ response show that there is not enough evidence-based knowledge on what to do with PRO results, e. g. there are only few to none reference manuals with thresholds for PROs [7]. Hence, our findings emphasize the need for the development of practical guidelines for clinicians for the routine use of PROs. For example, the principal investigators of the EDIUM study are planning to develop minimal important differences for both the EORTC QLQ-C30 and -CR29 using the results of EDIUM.

The authors are aware of some limitations resulting from the recruitment methods used: As clinicians from study centers nominated themselves if they were interested in participating, the presented study is biased by self-selection. However, the inconsistency and ambiguity of their statements highlight the variety of clinicians’ attitudes the study could carve out. The present study clearly has a small sample size, with only 12 participants. Although full content saturation cannot be claimed, the inclusion of different professions (physicians, nurses, psychologist) allows a more diverse view of the topic and hence conclusions that involve not just a single profession, but the whole CCC as a treatment team. This multidisciplinary approach may be regarded as a strength of the study. The comparison of different professional groups presented should not lead to any hasty conclusions being drawn and is only intended to show that more precise research questions need to be developed relative to the different views of PROs among the professions. For EDIUM, the findings form the basis of a quantitative questionnaire, the results of which will be reported elsewhere.

Although all the CCCs participating in this study are involved in the EDIUM study, not all of the interview partners had any initial experience with PROs yet. Some of them were therefore seeing a PRO questionnaire and its results for the first time during the interview. Some statements may thus be first impressions rather than well-founded opinions. Nevertheless, as implementation processes for PROs usually start in a setting in which there is generally little knowledge about PROs, these statements are also informative in revealing potential barriers to and prejudices against PROs.

In conclusion, the interview partners mostly showed a rather positive general attitude toward PROs, and this is an important first step toward using them clinically. Implementation strategies should take into account the advantages of PROs already mentioned by clinicians, without ignoring the reasonable doubts also expressed.

## Supplementary Information

**Additional file 1.**

**Additional file 2.**

## Data Availability

According to the patient consent form data is not available for scientific use by others than the project group members (contact: Nora Tabea Sibert, sibert@krebsgesellschaft.de).

## Footnotes

1 Further information about the EDIUM study is available at: http://edium-studie.de or https://innovationsfonds.g-ba.de/projekte/versorgungsforschung/edium-ergebnisqualitaet-bei-darmkrebs-identifikation-von-unterschieden-und-massnahmen-zur-flaechendeckenden-qualitaetsentwicklung.157 (both in German; last accessed January 25, 2021).

## References

[1] Fromme EK, Eilers KM, Mori M, Hsieh YC, Beer TM (2004). How accurate is clinician reporting of chemotherapy adverse effects? A comparison with patient-reported symptoms from the quality-of-life questionnaire C30. J Clin Oncol.

[2] Laugsand EA, Sprangers MA, Bjordal K, Skorpen F, Kaasa S, Klepstad P (2010). Health care providers underestimate symptom intensities of cancer patients: a multicenter European study. Health Qual Life Outcomes.

[3] Basch E, Deal AM, Kris MG, Scher HI, Hudis CA, Sabbatini P, et al. Symptom monitoring with patient-reported outcomes during routine cancer treatment: a randomized controlled trial. J Clin Oncol. 2015. 10.1200/JCO.2015.63.0830. 10.1200/JCO.2015.63.0830 PMC4872028 26644527

[4] Basch E, Deal AM, Dueck AC, Scher HI, Kris MG, Hudis C, Schrag D (2017). Overall survival results of a trial assessing patient-reported outcomes for symptom monitoring during routine Cancer treatment. JAMA..

[5] Denis F, Yossi S, Septans A-L, Charron A, Voog E, Dupuis O, Ganem G, Pointreau Y, Letellier C (2017). Improving survival in patients treated for a lung cancer using self-evaluated symptoms reported through a web application. Am J Clin Oncol.

[6] Klinkhammer-Schalke M, Steinger B, Koller M, Zeman F, Fürst A, Gumpp J (1990). Diagnosing deficits in quality of life and providing tailored therapeutic options: results of a randomised trial in 220 patients with colorectal cancer. Eur J Cancer Oxf Engl.

[7] Graham ID, Logan J, Harrison MB, Straus SE, Tetroe J, Caswell W, Robinson N (2006). Lost in knowledge translation: time for a map?. J Contin Educ Heal Prof.

[8] Stover AM, Haverman L, van Oers HA, Greenhalgh J, Potter CM, Ahmed S, et al. Using an implementation science approach to implement and evaluate patient-reported outcome measures (PROM) initiatives in routine care settings. Qual Life Res. 2020. 10.1007/s11136-020-02564-9. 10.1007/s11136-020-02564-9 PMC8528754 32651805

[9] Field B, Booth A, Ilott I, Gerrish K (2014). Using the knowledge to action framework in practice: a citation analysis and systematic review. Implement Sci IS.

[10] Devlin NJ, Appleby J, Buxton M (2010). King Edward’s Hospital Fund for London, Office of Health Economics London E. getting the most out of PROMs: putting health outcomes at the heart of NHS decision-making.

[11] Ajzen I (1991). The theory of planned behavior. Organ Behav Hum Decis Process.

[12] Eccles MP, Grimshaw JM, Johnston M, Steen N, Pitts NB, Thomas R, Glidewell E, Maclennan G, Bonetti D, Walker A (2007). Applying psychological theories to evidence-based clinical practice: identifying factors predictive of managing upper respiratory tract infections without antibiotics. Implement Sci IS..

[13] Ajzen I (1991). The theory of planned behavior. Organ Behav Hum Decis Process.

[14] Aaronson NK, Ahmedzai S, Bergman B, Bullinger M, Cull A, Duez NJ (1993). The European organization for research and treatment of cancer QLQ-C30: a quality-of-life instrument for use in international clinical trials in oncology. J Natl Cancer Inst.

[15] Whistance RN, Conroy T, Chie W, Costantini A, Sezer O, Koller M (2009). Clinical and psychometric validation of the EORTC QLQ-CR29 questionnaire module to assess health-related quality of life in patients with colorectal cancer. Eur J Cancer Oxf Engl 1990.

[16] Seufferlein T, Post S, Wesselmann S, Rückher J, German Cancer Society (DKG), Certification Committee Visceral Oncology Centres / Colorectal Cancer Centres (2018). Annual Report 2020 of the Certified Colorectal Cancer Centers (CRCCs). Audit year 2019 / Indicator.

[17] van der Hout A, Neijenhuijs KI, Jansen F, van Uden-Kraan CF, Aaronson NK, Groenvold M, Holzner B, Terwee CB, van de Poll-Franse LV, Cuijpers P, Verdonck-de Leeuw IM (2019). Measuring health-related quality of life in colorectal cancer patients: systematic review of measurement properties of the EORTC QLQ-CR29. Support Care Cancer.

[18] Scott NW, Fayers P, Aaronson NK, Bottomley A, de Graeff A, Groenvold M, Gundy C, Koller M, Petersen MA, Sprangers MAG (2008). EORTC QLQ-C30 Reference Values Manual.

[19] Kuckartz U (2014). Qualitative text analysis.

[20] Sprangers MA, Cull A, Bjordal K, Groenvold M, Aaronson NK (1993). The European Organization for Research and Treatment of Cancer. Approach to quality of life assessment: guidelines for developing questionnaire modules. EORTC study group on quality of life. Qual Life Res Int J Qual Life Asp Treat Care Rehabil..

[21] van der Wees PJ, Verkerk EW, Verbiest MEA, Zuidgeest M, Bakker C, Braspenning J, de Boer D, Terwee CB, Vajda I, Beurskens A, van Dulmen SA (2019). Development of a framework with tools to support the selection and implementation of patient-reported outcome measures. J Patient-Rep Outcomes.

[22] Sibert NT, Dieng S, Oesterle A, Feick G, Carl G, Steiner T, et al. Psychometric validation of the German version of the EPIC-26 questionnaire for patients with localized and locally advanced prostate cancer. World J Urol. 2019. 10.1007/s00345-019-02949-7. 10.1007/s00345-019-02949-7 31552467

[23] Klinkhammer-Schalke M, Koller M, Steinger B, Ehret C, Ernst B, Wyatt JC (2012). Direct improvement of quality of life using a tailored quality of life diagnosis and therapy pathway: randomised trial in 200 women with breast cancer. Br J Cancer.

[24] Rutherford C, Campbell R, White K, King M (2019). Patient-reported outcomes as predictors of survival in patients with bowel cancer: a systematic review. Qual Life Res Int J Qual Life Asp Treat Care Rehabil..

[25] Hsu T, Speers CH, Kennecke HF, Cheung WY (2017). The utility of abbreviated patient-reported outcomes for predicting survival in early stage colorectal cancer. Cancer..

[26] Davis FD (1989). Perceived usefulness, perceived ease of use, and user acceptance of information technology. MIS Q.

[27] HOLDEN RJ, B-T KARSH (2010). The technology acceptance MODEL: its past and its future in health care. J Biomed Inform.

[28] Foster A, Croot L, Brazier J, Harris J, O’Cathain A (2018). The facilitators and barriers to implementing patient reported outcome measures in organisations delivering health related services: a systematic review of reviews. J Patient-Rep Outcomes..

[29] Santana MJ, Haverman L, Absolom K, Takeuchi E, Feeny D, Grootenhuis M, Velikova G (2015). Training clinicians in how to use patient-reported outcome measures in routine clinical practice. Qual Life Res Int J Qual Life Asp Treat Care Rehabil.

