# Supplementary material for: Clinicians’ knowledge and attitudes towards patient reported outcomes in colorectal cancer care – insights from qualitative interviews
Source: BMC Health Serv Res. 2021 Apr 20;21:366. doi: 10.1186/s12913-021-06361-z (PMC8056693; doi:10.1186/s12913-021-06361-z)
Supplement: Supplementary file 1 — Additional file 1. [file 12913_2021_6361_MOESM1_ESM.docx]

**Title**: Clinicians’ knowledge and attitudes towards patient reported outcomes in colorectal cancer care – insights from qualitative interviews

**Authors**

1. Corresponding author:

Nora Tabea Sibert
German Cancer Society
Kuno-Fischer-Straße 8, 14057 Berlin, Germany
sibert@krebsgesellschaft.de; +49 30 322 932 968

1. Christoph Kowalski
   German Cancer Society
   Kuno-Fischer-Straße 8, 14057 Berlin, Germany

[kowalski@krebsgesellschaft.de](mailto:kowalski@krebsgesellschaft.de); +49 30 322 932 947

1. Holger Pfaff
   University of Cologne, Faculty of Human Sciences and Faculty of Medicine, Institute of Medical Sociology, Health Services Research and Rehabilitation Science

Eupener Str. 129, 50933 Köln, Germany
holger.pfaff@uk-koeln.de; +49(0)221 478-97100

1. Simone Wesselmann
   German Cancer Society

Kuno-Fischer-Straße 8, 14057 Berlin, Germany
[wesselmann@krebsgesellschaft.de](mailto:wesselmann@krebsgesellschaft.de); +49 30 322 932 990

1. Clara Breidenbach

German Cancer Society
Kuno-Fischer-Straße 8, 14057 Berlin, Germany
[breidenbach@krebsgesellschaft.de](mailto:breidenbach@krebsgesellschaft.de); +49 30 322 932 934

# Additional File 1: Interview Guide

## Part 1: Main interview guide

| 1 To start with, could you briefly describe your work in the hospital? | - Field of activity/position - Ward - Working hours - How long have you had the position? |
| --- | --- |
| 2 How is the EDIUM study going for you?  — Or, referring to PROs more directly: how do you collect PROs in the framework of the EDIUM study? | - Patient information - Clarifying wording (what do you call PROs / quality-of-life profiles?) - Collecting PROs / quality-of-life data - Participants |
| 3 In what ways are you using the PROs / quality-of-life profiles clinically?  *Or:*  What sort of role do PROs play in your routine clinical work? | - Inclusion in treatment planning - Preparation for discussions - In which specialty? (Specialist service, psychological, oncological, nursing) - Importance in everyday clinical work - Reasons for use / nonuse   - Access options   - Presentation   - Importance / meaningfulness   - Embedding in workflows |
| 4 What do you think about the PROs? | - Benefits for patients - Benefits for clinical staff - Meaningfulness (general — e.g., scientific) - Presentation of PROs in EDIUM - Optional access in EDIUM |
| 5 If you could decide, how would PROs be used in your routine clinical work? | - Time point of survey - Process implementation (time point, participants, access) - Presentation |

## Part 2 (show the interviewee three or four presentation options)

| 1 Which aspects of the presentation are important for you?  How should PROs be presented so that they’re easy for you to work with? | - Form (paper or digital) - Conciseness / length - Clarity - Completeness (only relevant scores?) - Information content (individual questions) - Color design - Reference values - Interpretation aids (difference between function scale and symptom scale) |
| --- | --- |
| 2 What do you think of these presentation styles? | - When you look at these presentation styles for PROs, which ones would you prefer to use clinically? - Why this one? Why not the others? |
| 3 Do you have any other suggestions for improvement? Would you like to say / add anything else? Do you think there are any aspects that haven’t yet been covered? | |

*Part 3: Sociodemographic information*

1. In which year were you born?

2. What is your gender?

□ male

□ female

□ divers

3. Which profession do you have?

□ nurse

□ with specializing in oncology

□ without specializing in oncology

□ physician

□ assistant physician

□ specialist

□ senior physician

□ chief physician

□ psycho-oncologist

□ social worker

□ other: _________________________
