# Supplementary material for: Clinicians’ knowledge and attitudes towards patient reported outcomes in colorectal cancer care – insights from qualitative interviews
Source: BMC Health Serv Res. 2021 Apr 20;21:366. doi: 10.1186/s12913-021-06361-z (PMC8056693; doi:10.1186/s12913-021-06361-z)
Supplement: Supplementary file 2 — Additional file 2. [file 12913_2021_6361_MOESM2_ESM.docx]

**Title**: Clinicians’ knowledge and attitudes towards patient reported outcomes in colorectal cancer care – insights from qualitative interviews

**Authors**

1. Corresponding author:

Nora Tabea Sibert
German Cancer Society
Kuno-Fischer-Straße 8, 14057 Berlin, Germany
sibert@krebsgesellschaft.de; +49 30 322 932 968

1. Christoph Kowalski
   German Cancer Society
   Kuno-Fischer-Straße 8, 14057 Berlin, Germany

[kowalski@krebsgesellschaft.de](mailto:kowalski@krebsgesellschaft.de); +49 30 322 932 947

1. Holger Pfaff
   University of Cologne, Faculty of Human Sciences and Faculty of Medicine, Institute of Medical Sociology, Health Services Research and Rehabilitation Science

Eupener Str. 129, 50933 Köln, Germany
holger.pfaff@uk-koeln.de; +49(0)221/478-97100

1. Simone Wesselmann
   German Cancer Society

Kuno-Fischer-Straße 8, 14057 Berlin, Germany
[wesselmann@krebsgesellschaft.de](mailto:wesselmann@krebsgesellschaft.de); +49 30 322 932 990

1. Clara Breidenbach

German Cancer Society
Kuno-Fischer-Straße 8, 14057 Berlin, Germany
[breidenbach@krebsgesellschaft.de](mailto:breidenbach@krebsgesellschaft.de); +49 30 322 932 934

# Additional File 2: Code Book

| **Category** | | | | | **Definition** | **Anchor example** |
| --- | --- | --- | --- | --- | --- | --- |
| **Wording** | | | | | 1. Answer to the guideline question: What do you call the questionnaire used for EDIUM? 2. Any particular wording participants use for PROs and/or the questionnaire used for EDIUM. | No anchor example needed |
|  | PRO/patient-reported outcomes | | | | No definition needed | No anchor example needed |
|  | Quality of life | | | |  |  |
|  | Scales/function and symptom scales | | | |  |  |
|  | Questionnaire | | | |  |  |
|  | No name | | | |  |  |
|  | Other (outcome, assessment tool, quality data) | | | |  |  |
| **General attitude toward PROs** | | | | | Statements that reflect the participants’ general attitude toward PROs.^[[1]](#footnote-1)^ |  |
|  | (Rather) positive attitude | | | | Statements that reflect a (rather) positive attitude toward PROs. | “I do think it’s useful, otherwise I wouldn’t take part in it, I’ve supported it.” (CH04f, paragraph 20) |
|  |  | Advantages of PROs | | | Statements that describe advantages of (using) PROs. |  |
|  |  |  | Standardization/quantification | | Statements that highlight the quantifying approach of PROs. | “Because in the end it’s a matter of standardization, so that things can be made comparable.” (CH01m, paragraph 20) |
|  |  |  | Additional information gained | | Statements that claim information can be gained by using PROs. | “And the questionnaire definitely also includes questions I wouldn’t necessarily ask routinely, so it definitely brings in aspects that might otherwise get lost.” (CH02f, paragraph 16) |
|  |  |  | Visualization of disease progression | | Statements that claim PROs can visualize disease progression. | “So you can observe some kind of course developing. Exactly — what was it like before? What’s it like in the middle? And then afterward?” (OFP01w, paragraph 34) |
|  |  |  | Outcomes are relevant for patients | | Statements that mention the relevance of PROs for patients. | “I think it’s very positive, because it’s relevant for the patients. I mean, so if we say we’ve had a curative treatment approach, then the patients will have to or may be able to live with the situation for another 10, 20, 30 years. And that needs to go along with a reasonable quality of life — and particularly with rectal cancer patients, both of us know that isn’t always the case.”(CH02f, paragraph 16) |
|  |  |  | Support for patient–clinician communications | | Statement that emphasize how PROs can support communications between clinicians and patients. | “And then you can also use it to offer a discussion or to arrange for someone who could then offer a discussion.” (IM01m, paragraph 20) |
|  |  | Possible example uses | | | Statements that mention possible example uses for PROs. |  |
|  |  |  | Clinical | | Statements that mention examples of possible clinical uses for PROs. | “I can imagine using this [the PRO instruments], or even having it included in treatment planning.” (CH01m, paragraph 24) |
|  |  |  |  | Screening tool | Statements mentioning that PROs could be used as a screening tool (e.g., for psycho-oncological needs, specialized support). | “If we weren’t seeing all of the patients personally, then I could imagine the questionnaire could also be quite useful for filtering out where we should go.” (PO01w, paragraph 16) |
|  |  |  |  | Treatment planning | Statements mentioning that PROs could be used for treatment planning. | “But I’d mainly be interested first of all in the questionnaire from beforehand, because from that you can quickly find out a lot of things you need to pay attention to, for treatment planning as well.” (IM01m, paragraph 16) |
|  |  |  |  | Treatment monitoring | Statements mentioning that PROs could be used for treatment monitoring. | “So of course you could pass this questionnaire back to the therapist, that would be an option.” (PA0102, paragraph 30) |
|  |  |  | Scientific | | Statements mentioning examples of possible scientific uses for PROs. | “That’s why I think the thing with the questionnaires is very, very good, and of course — and this is where the scientific approach comes in — you can also quantify it.” (CH02f, paragraph 16) |
|  |  |  | quality assurance | | Statements mentioning examples of possible quality-assurance uses for PROs. | “Because the whole medical market and patients care will use these, ones like this, these are different quality data from the purely key figures provided during the certification procedures, so to speak, and together it also produces a much more specific picture of the center.” (CH01m, paragraph 20) |
|  | (Rather) negative attitude | | | | Statements that reflect a (rather) negative attitude toward PROs. |  |
|  |  | Doubts about questionnaire | | | Statements casting doubt on the questionnaires used for EDIUM (EORTC QLQ-C30 and -CR29). |  |
|  |  |  | Unspecific scaling | | Statements mentioning that the EORTC QLQ-C30 and -CR29 scores are unspecific and/or inaccurate. | “And I think the scaling isn’t very detailed. I mean, it’s actually relatively rough in my view.” (PO01w, paragraph 40) |
|  |  |  | Unspecific questions | | Statements mentioning that the EORTC QLQ-C30 and -CR29 questions are unspecific and/or imprecise. | “Otherwise, I think the others are sometimes very unspecific and may not give you any specific help.” (CH04f, paragraph 22) |
|  |  |  | Questions not relevant for in-patient cancer care | | Statements mentioning that the EORTC QLQ-C30 and -CR29 questions are not relevant to in-house cancer care. | “Well, I think that’s a problem of course for an acute-care hospital like this one. Because of course the symptoms and problems here are so extremely varied, especially at the start … Of course, when the patients are back home and everything has settled down. I think then the whole thing here is much more important.” (PF01f, paragraph 10) |
|  |  |  | Questions too specific | | Statements mentioning that the EORTC QLQ-C30 and -CR29 questionnaires are too specific and/or too long. | “So in that sense for colorectal patients, I think it would actually be quite good to focus a bit on the typical symptoms, such as dry mouth, hair loss is maybe more to do with chemotherapy and not so much before the operation, that might be something I would tend to leave out. And maybe a few things could be summed up a bit. Pain here, for example, there are several forms here like abdominal pain, anal, rectal, and the incontinence stories could maybe tend to be summed up a bit.” (CH02f, paragraph 34) |
|  |  | Doubts about the need for PROs | | | Statements doubting the general need for PROs. |  |
|  |  |  | No additional information | | Statements indicating that no additional information is gained when using PROs. | “So it’s already a bit superfluous.” (PA0102, paragraph 30) |
|  |  |  | Cannot replace face-to-face conversation between clinicians and patients | | Statements indicating that PROs cannot replace face-to-face communication between clinicians and patients. | “Well, it’s always — when it’s about very specific, personal areas, I think, then it’s, for many patients it gets difficult, it’s the financial side on the one hand, where many people probably find it hard to make a statement, that’s probably better done in a personal conversation, I think.” (IM01m, paragraph 20) |

1. “Attitude” is used here in the sense as proposed by Ajzen, “The theory of planned behavior” (1991), as explained in the article. [↑](#footnote-ref-1)
